# Helicobacter-stimulated IL-10-producing B cells suppress differentiation of lipopolysaccharide/Helicobacter felis-activated stimulatory dendritic cells

**DOI:** 10.3906/biy-2012-11

**Published:** 2021-04-20

**Authors:** Doğuş ALTUNÖZ, Ayça SAYI YAZGAN

**Affiliations:** 1 Department of Molecular Biology and Genetics, Faculty of Science and Letters, İstanbul Technical University, İstanbul Turkey

**Keywords:** *Helicobacter felis*, regulatory B cells, dendritic cells, lipopolysaccharide

## Abstract

Regulatory B cells (Bregs) produce antiinflammatory cytokines and inhibits proinflammatory response. Recently, immunosuppressive roles of Bregs in the effector functions of dendritic cells (DCs) were demonstrated. However, cross talk between Bregs and DCs in *Helicobacter* infection remains unknown. Here, we showed that direct stimulation of bone marrow-derived DCs (BM-DCs) with *Helicobacter felis* (*H. felis*) antigen upregulates their CD86 surface expression and causes the production of interleukin-6 (IL-6), tumor necrosis factor alpha (TNF-α), interleukin-12 (IL-12), and interleukin-10 (IL-10). Furthermore, prestimulation of DCs with supernatants derived from both *Helicobacter*-stimulated IL-10^–^ B (*Hf*_*stim*_-IL-10^–^ B) or IL-10^+^ B (*Hf*_*stim*_-IL-10^+^) cells suppresses the secretion of TNF-α and IL-6, but does not affect the expression of CD86 and secretion of IL-12 by lipopolysaccharide (LPS) or *H. felis*-activated BM-DCs. Remarkably, soluble factors secreted by *Hf*_*stim*_-IL-10^–^ B cells, but not by *Hf*_*stim*_-IL-10^+^ B cells, suppress the secretion of IL-10 by BM-DCs upon subsequent LPS stimulation. In contrast, prestimulation with BM-DCs with supernatants of *Hf*_*stim*_-IL-10^+^ B cells before *H. felis *antigen stimulation induces significantly their IL-10 production. Collectively, our data indicated that prestimulation with soluble factors secreted by *Hf*_*stim*_-IL-10^+^ B cells, DCs exhibit a tolerogenic phenotype in response to LPS or *Helicobacter* antigen by secreting high levels of IL-10, but decreased levels of IL-6 and TNF-α.

## 1. Introduction

*Helicobacter pylori *(*H. pylori*) are gram-negative bacteria, which multiplies in human stomach. It has previously demonstrated that gastric biopsies from persistently *H. pylori*-infected individuals have more infiltrated innate and adaptive immune cells compared to uninfected individuals (Moyat and Velin, 2014). 

Dendritic cells (DCs) have critical roles in effector and tolerogenic immune response in *H. pylori *within the stomach as antigen-presenting cells (Peek et al., 2010). *H. pylori *infection upregulates IL-1α, IL-6, IL-1β, and IL-23p19 mRNA expressions in bone marrow-derived DCs (BM-DCs) (Kao et al., 2006; Horvath et al., 2012). BM-DCs also produce the antiinflammatory cytokine IL-10 in response to *H. pylori* infection (Rizzuti et al., 2015). Furthermore, *H. pylori*-pulsed human monocyte-derived DCs upregulate MHC-II, CD80, CD86, and CD83 surface expression and secrete TNFα, IL-6, IL-10, and IL-12 cytokines (Hafsi et al., 2004; Hoces de la Guardia et al., 2013; Käbisch et al., 2014; Käbisch et al., 2016). Alternatively, the tolerogenic DCs regulate effector T cell responses in *Helicobacter* infection. It was shown that live *H. pylori* suppresses maturation of DCs and their IL-6 and IL-12 production, which are efficiently induced by lipopolysaccharide (LPS) (Jiang et al., 2002; Oertli et al., 2012). In addition, the depletion of DCs in mice results in abrogation of *H. pylori-*driven immune tolerance and promotes T cell-mediated immunological pathology (Oertli et al., 2012). *H. pylori*-infected BM-DCs induce regulatory T cells (Tregs) and decrease Th17/Treg ratio in vitro (Kao et al., 2010; Zhang et al., 2010; Oertli et al., 2012). *Helicobacter felis* (*H. felis*) is gram-negative bacteria, which belong to *Helicobacter* species with a higher immunogenicity than *H. pylori* in mice (Sayi et al., 2009). Previously, it was reported that *H. felis *antigen induces DCs to secrete IL-6 and IL-10 (Drakes et al., 2006). 

A complex cross talk exists between B cells and DCs. B cells regulate maturation and differentiation of monocyte-derived DCs upon BCR and TLR9 (CpG) induction (Maddur et al., 2012). CpG- and CD40L-activated B cells have an inhibitory role in IL-12p70 production in mature DCs through cellular contact and soluble factors (Morva et al., 2012). Regulatory influence of B cells on DCs have been demonstrated in *Leishmania major *infection. *L. major*-stimulated B cells inhibit IL-12 production by DCs in an IL-10-dependent manner (Ronet et al., 2010). Soluble factors from *Escherichia coli*-stimulated regulatory B cells inhibit maturation and inflammatory properties of BM-DCs by modulating their CD86 expression and TNF-α production (Maerz et al., 2020). It has been previously reported that supernatant derived from IL-10-producing CD138^+^ plasmablasts, but not from *IL10*^*–/– *^ plasmablasts leads to a significant decrease in IL-6 and IL-12 mRNA expression in DCs (Matsumoto et al., 2014).

The immunosuppressive and protective roles of regulatory B cells (Bregs) in *Helicobacter* infection have been demonstrated. *H. felis*-stimulated B cells cause differentiation of the T regulatory-1 (Tr1) cells (Sayi et al., 2011). Recently, we have elucidated roles of *H. felis*-stimulated IL-10-producing B (*Hf*_*stim*_-IL-10^+^ B) and IL-10-nonproducing B (*Hf*_*stim*_-IL-10^–^ B) cells in the Tr1 differentiation. It has been demonstrated that *Hf*_*stim*_-IL-10^+^ B cells secrete IL-10, whereas *Hf*_*stim*_-IL-10^–^ B cells secrete IL-6, TGF-β, and TNF-α cytokines. Furthermore, both *H. felis*-stimulated IL-10^–^ B cells and IL-10^+^ B cells induce differentiation of Tr-1 cells. Besides, *Hf*_*stim*_-IL-10^–^ B cells secrete IL-6 and TGF-βthat cause Th17-cell differentiation (Said et al., 2018). However, up until now, the influence of B cells on immature BM-DCs in *Helicobacter* infection remain to be elucidated. In this study, we evaluated (i) direct effect of *H. felis* antigen (ag), (ii) the effect of soluble factors secreted by *H. felis*-stimulated IL-10^+^ B cells and IL-10^–^ B cells on the activation status and cytokine profile of immature BM-DCs in vitro.

Here we demonstrated that direct stimulation of immature BM-DCs with *Helicobacter* ag induces upregulation of CD86 surface expression and production of TNF-α, IL-6, IL-12, and IL-10, similar to the effect of *E. coli* LPS. Moreover, we found that conditioned medium from *H. felis*-stimulated IL-10^+^ B and IL-10^–^ B cells induces DCs to upregulate surface expression of CD86 and to secrete high levels of IL-12. Furthermore, subsequent stimulation of DCs with LPS or *Helicobacter *ag leads to suppression in the secretion of IL-6 and TNF-α. Finally, we concluded that DCs secreted high levels of IL-10, upon prestimulation with soluble factors of *Hf*_*stim*_-IL-10^+^ B cells, before LPS or *Helicobacter* stimulation.

## 2. Materials and methods

### 2.1. Animal experiments

We obtained C57BL/6 mice from Boğaziçi University, Vivarium (İstanbul, Turkey) and kept them under pathogen-free conditions. We performed all experiments in accordance with the guidelines, which were approved by the Boğaziçi University Local Ethical Committee on Animal Experiments. 

### 2.2. Bacteria

We received* H. felis* from Prof. Anne Müller (University of Zurich, Zurich, Switzerland). We grow *H. felis* as previously described (Sayi et al., 2009). We performed bacterial sonication on a Bandelin Sonopuls (30 s pulse-on; 50 s pulse-off for 6’30”, 50 Won ice). We determined the protein concentration of *H. felis* antigens using BCA Protein Kit (Thermo Fisher Scientific, Munich, Germany).

### 2.3. Generation of bone marrow-dendritic cells (BM-DCs) 

We isolated bone marrow cells from the femur bone of C57BL/6 mice and differentiated into BM-DCs as explained previously, with some modifications (Matheu et al., 2008). Briefly; we seeded 25 × 10^4^ bone marrow cells per well in 96-well plates in the DC culture media Roswell Park Memorial Institute (RPMI) medium containing 1% pen-strep, 10% fetal bovine serum (FBS), 10 ng/mL recombinant granulocyte-macrophage colony-stimulating factor (rGM-CSF; BioLegend, San Diego, CA,USA), 10 ng/mL recombinant interleukin-4 (rIL-4; Gibco) and 50 μM beta-mercaptoethanol). Every 2 days, we refreshed half of the media with freshly prepared DC culture media. DCs were differentiated after 7 days and cells were collected using PBS containing 10 mM EDTA. Then, for enriching CD11c+ DCs, we performed magnetic separation using anti-CD11c microbeads, following manufacturer’s instructions (Miltenyi Biotec, Bergisch Gladbach, Germany). After that, we cultured 5 × 10^4^ separated DCs per well in 96-well plates in RPMI medium containing 10% FBS and 1% pen-strep. We determined purity of BM-DCs based on the expression of CD11c by flow cytometry (BD Accuri C6Flow Cytometer, Accuri Cytometers, Ann Arbor, MI, USA).

### 2.4. Isolation and stimulation of B cells

We collected spleens from C57BL/6 mice and isolated CD19^+^ B cells from a single-cell suspension of spleens by immunomagnetic separation following manufacturer’s instructions (m-B Cell Isolation Kit, Miltenyi Biotec). We stained splenic B cells with CD19-FITC antibody (Biolegend) to determine the purity of cells. Afterwards, we cultured B cells in 96-well round-bottom plates in RPMI containing 1% pen-strep and 10% (FBS) as 5 × 105 cells/well and induced with 5 ug/mL *H. felis* antigen for 24 h. During the last 5 h of incubation, we induced cells with ionomycin (500 ng/mL) and phorbol 12-myristate 13-acetate (50 ng/mL). Later, we isolated IL-10^+^ B and IL-10^–^ B cells from *Helicobacter felis*-stimulated B cells (*Hf*_*stim*_-B) by immunomagnetic separation following manufacturer’s instructions (m. Reg. B Cell Isolation Kit, Miltenyi Biotec).

### 2.5. Stimulation of immature BM-DCs with H. felis antigen or the conditioned medium of B cell subsets 

We treated IL-10^–^ B and IL-10^+^ B cells with 5 μg/mL *H. felis* antigen for 8 h. We cultured immature DCs in fresh medium without stimulation or in the supernatant of IL-10^+^ B or IL-10^–^ B cells for 24 h. Afterwards, we washed DCs and treated with *H. felis* antigen (10 µg/mL) or LPS (100 ng/mL) for 12 h or left untreated.

### 2.6. ELISA

We measured TNF-α, IL-12/IL-23 (p40), IL-6, and IL-10 secretion using m-IL-12/IL-23 (p40) ELISA kit (431606; Biolegend), m-TNF-α ELISA kit (430906; Biolegend), m-IL-6 ELISA kit (431301; Biolegend), and m-IL-10 ELISA kit (431416; Biolegend), respectively, following manufacturer’s instructions (Biolegend). ELISA detection was done using a microplate reader (Benchmark Plus reader, Bio-Rad Laboratories, Hercules, CA, USA) at 450 nm.

### 2.7. Flow cytometry

We detached BM-DCs from the plates through incubation for 10 min with PBS containing 10 mM EDTA. We used following antimouse antibodies to detect following markers: anti-CD86-PE (clone GL-1), anti-CD11c-APC (clone N418), CD86 (clone GL-1), anti-CD19-FITC (clone 6D5). All antibodies were obtained from Biolegend. For staining, we incubated cells in PBS containing 2% FBS together with antibodies for 1 h on ice. We performed flow cytometry on a Becton Dickinson (BD) ACCURI C6 (BD Biosciences, San Jose, CA, USA) machine and analyzed using FlowJo software.

### 2.8. Statistical analysis

We used GraphPad Prism 6 for statistical analyses. Standard error of the mean (SEM) was shown in column bar graphs. We calculated the p-values by two-tailed Student’s t-test, and we considered p < 0.05 as statistically significant (ns: not significant).

## 3. Results 

### 3.1. H. felis antigen-activated BM-DCs

To assess the direct effect of *H. felis* antigen (ag) on immature DCs, we differentiated bone-marrow cells into bone marrow-derived DCs (BM-DCs) by adding 10 ng/mL rGM-CSF and 10 ng/mL rIL-4 over the course of 7 days. We then enriched CD11c+ DC population using immunomagnetic sorting. The purity of BM-DCs before and after immunomagnetic sorting was around 72% and 91%, respectively (Figures 1A and 1B). Next, we evaluated the activation/maturation status of *H. felis*-induced BM-DCs through their CD86 expression. We also treated BM-DCs with LPS as it provides maturation and activation signals to DCs. Flow cytometric analysis demonstrated that stimulation of immature DCs with either LPS or *H. felis* ag for 12 h induced similar expression of CD86 (B7.2) (Figures 1C and 1D). These data revealed that *H. felis* antigen, opposite of *H. pylori* leads to high expression of CD86, which is a determinant of DC maturation, in an in vitro setting. 

### 3.2. H. felis antigen-induced secretion of both pro- and antiinflammatory cytokines by BM-DCs

Previously, it was shown that treatment of BM-DCs with *H. felis *ag for 3 days, induces the production of IL-6 with small amounts of IL-10 (Drakes et al., 2006). To investigate the detailed cytokine profile of *H. felis* antigen-stimulated BM-DCs (*Hf*_*stim*_-BM-DCs), we treated BM-DCs with *H. felis* antigen for 12 h. We detected elevated secretion levels of both antiinflammatory (IL-10) and proinflammatory (TNF-α, IL-12, and IL-6) cytokines in *Hf*stim-BM-DCs, similar to LPS (Figures 2A–2D). These data indicate that *H. felis* antigenic proteins induce both pro- and antiinflammatory cytokine secretion from BM-DCs.

### 3.3. Suppression of IL-6, TNF-α, and IL-10 secretion in Hfstim-IL-10- B cells-conditioned medium preexposed LPS-stimulated BM-DCs 

Therefore, we asked whether *Hf*_*stim*_-IL-10^+^ B cells and *Hf*_*stim*_-IL-10^–^ B cells conditioned medium can affect activation and differentiation of BM-DCs. To address this point, we first incubated splenic B cells with 5 µg/mL *H. felis* ag for 24 h. Then, we separated IL-10^+^ B and IL-10^–^ B cells with more than 85% purity (data not shown). We treated both IL-10^+^ B and IL-10^–^ B cells with 5 μg/mL *H. felis* antigen for 8 h. We collected the cell-free supernatant and incubated immature BM-DCs with this conditioned medium before LPS or *H. felis* ag stimulation. Flow cytometric analysis of CD86 expression in DCs showed that conditioned medium from both *Hf*_*stim*_-IL-10^+^ B cells and *Hf*_*stim*_-IL-10^–^ B cells was efficiently induced the activation and maturation of DCs (53.85% and 55.65%, respectively). Also, we detected preincubation of DCs with conditioned medium leads to a slight increase in CD86 expression compared to only LPS-stimulation (Figures 3A and 3B). 

Later, we analyzed the supernatants of BM-DCs for IL-12/IL-23 (p40), IL-6, TNF-α, and IL-10 production in the presence or absence of *Hf*_*stim*_-IL-10^+^ B cells- or *Hf*_*stim*_-IL-10^–^ B cells-conditioned media. Both of these conditioned medium induced high levels of IL-12/IL-23 (p40) secretion, but low levels of TNF-α, IL-6, and IL-10 secretion from BM- DCs (Figures 4A–4D). In case of preexposure of BM-DCs to both conditioned media before LPS or *H. felis* stimulation have a little effect on the level of IL-12 secretion from these cells (Figure 4A). However, TNF-α and IL-6 production were significantly lower when BM-DCs were preexposed to *Hf*_*stim*_-IL-10^+^ B cells or *Hf*_*stim*_-IL-10^–^ B-conditioned media before LPS stimulation (Figures 4B and 4C). Alternatively, IL-10 secretion was significantly decreased in LPS-treated BM-DCs, which had previously been exposed to the conditioned medium of *Hf*_*stim*_-IL-10^–^ B cells. Of note, *Hf*_*stim*_-IL-10^+^ B cells-conditioned medium did not have such an inhibitory influence on IL-10 secretion from BM-DCs, which were later stimulated by LPS (Figure 4D). 

### 3.4. Decreased in IL-6 and TNF- α, but increased in IL-10 secretion by H. felis stimulated BM-DCs, which were preexposed to Hfstim-IL-10^+^ B cells-conditioned medium

To assess how preexposure to *Hf*_*stim*_-IL-10^+^ B cells and *Hf*_*stim*_-IL-10^–^ B cells-conditioned medium affect response of BM-DCs to *H. felis* antigen stimulation, we cultured BM-DCs in the presence of *Hf*_*stim*_-IL-10^+^ B cells or *Hf*_*stim*_-IL-10^–^ B cells conditioned medium for 24 h. Similar to LPS stimulation, level of CD86 expression was induced slightly upon prestimulation of DCs with *Hf*_*stim*_-IL-10^+^ B cells- or *Hf*_*stim*_-IL-10^–^ B cells-conditioned medium before *H. felis *stimulation compared to only *H. felis*-stimulated DCs (Figures 3A and 3B). Moreover, DCs produced less TNF-α and IL-6 when they were preexposed to *Hf*_*stim*_-IL-10^+^ B cells or *Hf*_*stim*_-IL-10^–^ B cell-conditioned medium before *H. felis* stimulation compared to only *H. felis*-stimulated DCs; however, no significant change in IL-12 production were detected (Figures 4A–4C). In addition, *Hf*_*stim*_-IL-10^+^ B cells-conditioned medium, but not *Hf*_*stim*_-IL-10^–^ B cells-conditioned medium, induced IL-10 production by DCs (Figure 4D). In conclusion, our results revealed the ability of indirect effects of *Hf*_*stim*_-IL-10^+^ B cells on conversion of DCs into semimature-like phenotype.

## 4. Discussion

Immune regulatory characteristics of B cells have been demonstrated in B cell-deficient mice, which cannot be recovered from autoimmune encephalitis due to lack of B cells (Wolf et al., 1996). Later, it was reported that B cell-derived IL-10 has crucial roles to suppress inflammation and induce Treg differentiation in mice (Fillatreau et al., 2002; Carter et al., 2011; Carter et al., 2012). Moreover, immune-suppressive functions of Bregs with IL-10 secretion in colitis (Mizoguchi et al., 2002), EAE (Fillatreau et al., 2002), arthritis (Mauri et al., 2003) and *Helicobacter* infection (Sayi et al., 2011) have been demonstrated. It has also been reported that *Helicobacter*-associated Bregs have a protective role in acute and chronic colitis (Li et al., 2019). Recently,we have shown that, *Hf*_*stim*_-IL-10^–^ B cells secrete IL-6, TGF-β, TNF-α, IgM, and IgG2b, whereas *Hf*_*stim*_-IL-10^+^ B cells produce IL-10 (Said et al., 2018). Bregs suppress the effector functions of DCs through IL-10 and indirectly prevent DC-mediated Th1 and Th17 differentiation (Sun et al., 2005; Matsumoto et al., 2014). 

Drakes et al*.* (2006) showed that 3 days’ stimulation with *H. felis* antigen (ag) leads to IL-6 and low levels of IL-10 secretion from murine BM-DCs. We contributed to this knowledge by showing that *H. felis* antigen can activate immature BM-DCs already after 12 h and can induce the secretion of IL-12 and TNF-α, in addition to IL-6 and IL-10, from these cells. *H. pylori* infection induces human monocyte-derived DCs obtained from PBMCs to upregulate the expression of costimulatory molecules and to secrete IL-12, TNF-α, IL-6, and IL-10 (Hafsi et al., 2004; Hoces de la Guardia et al., 2013; Käbisch et al., 2014; Käbisch et al., 2016). Alternatively, it was shown that *H. pylori *infection induces DC tolerization (Oertli et al., 2012; Oertli et al., 2013; Rizzuti et al., 2015). *H. pylori *infection did not cause maturation of murine BM-DCs, contrary to the effects of LPS. Also, *H. pylori*-infected murine BM-DCs secrete lower amounts of IL-12p40 and IL-6, but higher amounts of IL-10 compared to LPS-treated BM-DCs (Oertli et al., 2012). The different response of BM-DCs to *H. felis* versus *H. pylori *might be due to the effect of virulence factors in the latter one. 

Soluble factors secreted from B cells modulate characteristics and function of DCs (Ronet et al., 2010; Morva et al., 2012; Matsumoto et al., 2014; Maerz et al., 2020). Soluble factors secreted from CpG-ODN-stimulated B cells and LPS-stimulated IL-10-proficient-plasmablasts suppress IL-12 expression derived from human monocyte- derived mature DCs and mouse BM-DCs, respectively (Morva et al., 2012; Matsumoto et al., 2014). However, in our study, we detected soluble factors secreted from *Hf*_*stim*_-IL-10^+^ B or *Hf*_*stim*_-IL-10^–^ B cells increasing IL-12 secretion from murine BM-DCs. Also, these soluble factors can activate immature murine BM-DCs, detected by production of CD86. However, little secretion of IL-6, IL-10, and TNF-α were detected in these BM-DCs in the given conditions. The discrepancy between our results with the literature regarding IL-12 can be due to the influence of *H. felis* antigens in conditioned medium derived from *Hf*_*stim*_-IL-10^+^ B or *Hf*_*stim*_-IL-10^–^ B cells. *H. felis* antigens per se can induce the activation and IL-12 secretion of BM-DCs (Figure 2). Interestingly, Morva et al.’s (2012) data are in agreement with our IL-12 findings, as it reflects that even though the intracellular IL-12 in human mature DCs was significantly decreased when cocultured with CpG- ODN activated B cells and their supernatants, concentration of IL-12 was increased in supernatants of B-DC cocultured cells.

IL-10 derived from B cells inhibits the secretion of IL-12 by murine BM-DCs in response to stimulation with *L. *major, in an in vitro condition (Ronet et al., 2010). Secreted factors from *Hf*_*stim*_-IL-10^–^ B cells but not *Hf*_*stim*_-IL-10^+^ B cells can suppress the production of IL-10 by LPS-stimulated-BM-DCs. This may be due to the suppressive influence of proinflammatory TNF-α and IL-6 cytokines in supernatants of *Hf*_*stim*_-IL-10^–^ B cells on IL-10 production from BM-DCs (Banchereau et al., 2000; Said et al., 2018). Inversely; IL-10 production by *Helicobacter*-stimulated DCs was induced by pretreatment with soluble factors secreted from *Hf*_*stim*_-IL-10^+^ B cells. 

The elevated level of TNF-α secreted from DCs is linked to human autoimmune diseases such as IBD and psoriasis (Lowes et al., 2005; Baumgart et al., 2011). Remarkably, conditioned media from both *Hf*_*stim*_-IL-10^+^ B and *Hf*_*stim*_-IL-10^–^ B cells reduce the production of TNF-α by DCs. However, further studies are necessary to elucidate whether *Hf*_*stim*_-IL-10^+^ B and *Hf*_*stim*_-IL-10^–^ B cells suppress TNF-α production by DCs through similar mechanisms. It has been demonstrated that the differentiation of murine BM-DCs and human monocyte-derived DCs in the presence of TGF-β1 prevents the maturation of DCs in response to LPS stimulation (Mou et al., 2004; Fogel-Petrovic et al., 2007). Furthermore, the regulatory role of TGF-β on immature human DCs has been previously demonstrated (Adnan et al., 2016). Therefore, *Hf*_*stim*_-IL-10^–^ B cells might be responsible for suppressing the TNF-α production by DCs with the help of their TGF-β secretion.

Even though conditioned medium obtained from *Hf*_*stim*_-IL-10^+^ B or *Hf*_*stim*_-IL-10^–^ B also contains *H. felis* ag, we think that the regulatory effect of these conditioned medium on BM-DCs is not due to *Helicobacter* ag per se. Because, the amounts of IL-6, TNF-α, and IL-10 cytokines secreted from BM-DCs, which were kept in the conditioned medium derived from *Hf*_*stim*_-IL-10^+^ B or *Hf*_*stim*_-IL-10^–^ B cells without further LPS or *H. felis* ag stimulation, was far less than BM-DCs treated with only *H. felis* ag. Nevertheless, small amounts of *H. felis* ag remained in the supernatants secreted by *Hf*_*stim*_-IL-10^+^ B and *Hf*_*stim*_-IL-10^–^ B cells might be responsible for the secretion of these cytokines at low levels. Recent studies indicate that IL-10-producing, but not IL-10-nonproducing B cells regulate cytokine profile of DCs to indirectly inhibit T effector cell formation (Ronet et al., 2010; Matsumoto et al., 2014). Moreover, it is known that IL-10 inhibits activation and proinflammatory characteristics of DCs (Takenaka and Quintana, 2017). However, IL-6 has an important function in the Th17 cell differentiation together with TGF-β (Bettelli et al., 2006; Mangan et al., 2006; Veldhoen et al., 2006). It has been previously shown that plasmablast-derived IL-10 suppresses IL-6 mRNA expression in DCs and their function to induce the development of TGF-β- mediated Th17 cells (Matsumoto et al., 2014). Recently, it has been reported that *Escherichia coli*-stimulated regulatory B cells inhibit the production of TNF-α by BM-DCs in an IL-10-dependent manner (Maerz et al., 2020). In line with these findings, we here show that *Hf*_*stim*_-IL-10^+^ B cells can direct DCs to tolerogenic phenotype by modulating their IL-10, IL-6, and TNF-α production. However, it remains to be clarified in further studies whether DCs, prestimulated with the conditioned medium from *Hf*_*stim*_-IL-10^+^ B or *Hf*_*stim*_-IL-10^–^ B cells can induce regulatory T cell types or suppress Th-1- or Th-17-mediated effector immune responses. Collectively, prestimulation of DCs with *Hf*_*stim*_-IL-10^+^ B cells generate CD86+IL-12+IL-10^+^TNFloIL-6lo BM-DCs, which is reminiscent of semimature tolerogenic DC phenotype (Rutella et al., 2006).

In conclusion, this study demonstrates that *H. felis* ag directly stimulates BM-DCs to secrete proinflammatory cytokines and IL-10. Furthermore, *Hf*_*stim*_-IL-10^+^ B cells-derived soluble factors are responsible for generating IL-10-producing DC phenotype with reduced TNF-α and IL-6 production upon antigenic stimulation. Therefore, our study indicates that the regulatory interaction between B cells and DCs during *Helicobacter* infection contributes to the survival of the bacteria and prevention of symptoms in *Helicobacter*-infected individuals. It also emphasizes the versatile role of Bregs to prevent *Helicobacter*-associated immunopathology through interaction with an innate immune cell; DCs (Sayi et al., 2011). 

## Contribution of authors

DA performed all experiments and analyzed the data, and wrote the text. ASY designed and supervised the experiments and data analysis and edited the text. All authors have approved the final version of the manuscript.
